# Amino acid changes in the spike protein of feline coronavirus correlate with systemic spread of virus from the intestine and not with feline infectious peritonitis

**DOI:** 10.1186/1297-9716-45-49

**Published:** 2014-04-25

**Authors:** Emily Porter, Séverine Tasker, Michael J Day, Ross Harley, Anja Kipar, Stuart G Siddell, Christopher R Helps

**Affiliations:** 1 School of Cellular and Molecular Medicine, University of Bristol, Bristol BS8 1TD, UK; 2 School of Veterinary Sciences, University of Bristol, Langford, Bristol BS40 5DU, UK; 3 Veterinary Pathology, School of Veterinary Science, University of Liverpool, Leahurst Campus, Neston CH64 7TE, UK; 4 Present address: Institute of Veterinary Pathology, Vetsuisse Faculty, University of Zurich, Winterthurer Strasse 268, 8057 Zurich, Switzerland

## Abstract

Recent evidence suggests that a mutation in the spike protein gene of feline coronavirus (FCoV), which results in an amino acid change from methionine to leucine at position 1058, may be associated with feline infectious peritonitis (FIP). Tissue and faecal samples collected post mortem from cats diagnosed with or without FIP were subjected to RNA extraction and quantitative reverse-transcriptase polymerase chain reaction (qRT-PCR) to detect FCoV RNA. In cats with FIP, 95% of tissue, and 81% of faecal samples were PCR-positive, as opposed to 22% of tissue, and 60% of faecal samples in cats without FIP. Relative FCoV copy numbers were significantly higher in the cats with FIP, both in tissues (*P* < 0.001) and faeces (*P* = 0.02). PCR-positive samples underwent pyrosequencing encompassing position 1058 of the FCoV spike protein. This identified a methionine codon at position 1058, consistent with the shedding of an enteric form of FCoV, in 77% of the faecal samples from cats with FIP, and in 100% of the samples from cats without FIP. In contrast, 91% of the tissue samples from cats with FIP and 89% from cats without FIP had a leucine codon at position 1058, consistent with a systemic form of FCoV. These results suggest that the methionine to leucine substitution at position 1058 in the FCoV spike protein is indicative of systemic spread of FCoV from the intestine, rather than a virus with the potential to cause FIP.

## Introduction

Feline coronavirus (FCoV) infection is ubiquitous in domestic cats, particularly in multi-cat households where up to 90% of animals may be infected [1-3]. The majority of FCoV infections are asymptomatic or are associated with mild enteric disease [4]. However, approximately 5-12% of infected cats develop the invariably fatal disease, feline infectious peritonitis (FIP) [5-7].

One of the most important questions in FCoV research is why some FCoV-infected cats develop FIP, whereas others remain healthy. One current model of FIP pathogenesis proposes that cats are infected with FCoV by the faecal-oral route. Subsequently, the virus mutates into the virulent form. This form has an enhanced tropism for monocytes/macrophages, and in vitro studies suggest that this is reflected as sustainable replication in, and subsequent activation of, monocytes [8,9]. These activated monocytes carry the virus in the blood and, as a result of complex interactions with endothelial cells, induce the granulomatous phlebitis that is the pathogenic hallmark of FIP [10,11]. The age, breed, gender, reproductive status and immune response of individual cats also influence the development of FIP [12].

Currently, there is intense interest in determining which mutations alter the virulence of FCoVs. A recent paper published by Chang et al. [13] derived full genome sequence data from a collection of FCoVs obtained from the faeces of healthy cats and from the tissues of cats diagnosed with FIP. They provided evidence of an association between FCoV virulence and an amino acid substitution (methionine to leucine at position 1058, M1058L) within the putative fusion peptide of the FCoV spike (S) protein. Specifically, the authors concluded that the M1058L substitution distinguished FIP from non-FIP associated FCoVs in 92% of cases. A second substitution, two amino acids downstream of M1058L (serine to alanine at position 1060, S1060A) distinguished a further 4% of FIP from non-FIP associated FCoVs. The S protein fusion peptide is a critical element in the fusion of viral and cellular membranes during virus entry [14] and it is reasonable to think that amino acid substitutions within this peptide may alter the tropism of the virus.

In addition, a study by Licitra et al. has shown that it is possible to distinguish between FCoVs from animals with and without FIP on the basis of one or more substitutions in the amino acid sequence that comprises the furin cleavage motif within the S protein [15]. This furin cleavage site (consensus motif R-X-K/R-R, where R is the basic arginine residue, X is any residue and K is the basic lysine residue) delineates the border of the receptor-binding (S1) and fusion (S2) domains of the S protein and is distinct to the M1058L substitution site described above. Mutation at this site is proposed to alter proteolytic cleavage of the S protein and modify S protein fusogenic properties, which again may relate to the tropism of the virus [15].

Finally, Pedersen et al. [16] concluded that truncating and non-truncating mutations in the 3c gene occur in a significant proportion of FCoVs associated with FIP. Chang et al. [17] suggest that functional 3c protein expression is crucial for FCoV replication in the gut but is dispensable for systemic replication. However, they also do not exclude the possibility that the loss or alteration of the 3c protein may enhance the fitness of the virus in the monocyte/macrophage environment.

Over the past 8 years, the University of Bristol has collected a large number of post-mortem tissue and faecal samples from a cohort of thoroughly examined cats. These include cats with a definite diagnosis of FIP, confirmed by the presence of the typical histological FIP lesions, in which immunohistochemistry (IHC) demonstrated FCoV antigen within macrophages [18], and cats with diseases other than FIP that completely lacked any histological changes consistent with FIP. Importantly, this long-term study has enabled both faecal and tissue samples to be collected from FCoV-infected cats with and without FIP, allowing comparable samples from naturally infected cats to be examined. Samples were screened for FCoV RNA by quantitative reverse transcriptase-polymerase chain reaction (qRT-PCR) [19] and, if positive, were assessed for the M1058L substitution by pyrosequencing.

## Materials and methods

### Sample collection and storage

Post-mortem tissue samples, and faeces whenever possible, were collected from cats that were euthanized with suspected FIP, or due to other diseases. FIP was then definitively diagnosed or excluded by histopathology and, in the case of FIP, the demonstration of FCoV antigen in FIP lesions by IHC [18]. Tissues of cats without FIP were also tested by IHC for the presence of viral antigen.

Tissue samples were collected into RNAlater (Life Technologies) within 2 h of death for subsequent molecular analysis. The tissue samples were left in RNAlater for 24-48 h at room temperature or 4 °C before the RNAlater was discarded, and the tissue samples stored at -80 °C. The faecal samples were stored at -80 °C until use. Further samples were collected into 10% neutral-buffered formalin for histology and IHC. The tissues collected comprised primarily mesenteric lymph node, liver, kidney, spleen and omentum, while other tissues (e.g. intestine, brain, lung, pericardium, pancreas or other lymph nodes) were included based on gross pathological findings or reported clinical signs.

### Histology and IHC for FCoV antigen

The formalin-fixed tissue samples were subjected to standard processing for histopathology. They were embedded in paraffin wax and sections prepared and stained by haematoxylin-eosin. Sections were examined by a board-certified veterinary pathologist (MJD) at the University of Bristol for histopathological changes. Selected wax blocks were then sent to Veterinary Laboratory Services, University of Liverpool for IHC analysis as previously described [18]. For a cat to be assigned to the “FIP group”, it needed to have histopathological changes consistent with FIP in which FCoV antigen was demonstrated within macrophages in lesions [18]. For a cat to be assigned to the “non-FIP group”, histopathological changes consistent with FIP needed to be completely absent, and FCoV antigen within macrophages needed to be absent for all tissues. Only individual tissue samples with a positive qRT-PCR result, and lesions consistent with FIP on histopathology were used for pyrosequencing. Faecal samples were classified on the basis of the diagnosis attributed to the cat from which the sample originated.

### RNA extraction and qRT-PCR

Total RNA was extracted from 20 mg of tissue or 10 mg of faeces with a NucleoSpin RNA II kit (Macherey-Nagel) using methods based on previous work by Dye and Siddell [19,20]. Reverse transcription was done using a MJ Mini Gradient Thermal Cycler and ImProm II Reverse Transcriptase (Promega). Nine microlitres of total RNA solution were combined with 4 μL ImProm II 5× Reaction Buffer, 2.4 μL 25 mM MgCl_2_, 1 μL dNTPs (10 mM each), 1 μL random hexamers (0.5 μg/μL) and 1 μL ImProm II reverse transcriptase. The reaction was made up to a total volume of 20 μL with RNase-free water. The following thermal profile was used; 20 °C for 5 min, 42 °C for 30 min, 70 °C for 15 min and 4 °C hold. The resulting 20 μL of cDNA was added to 30 μL of RNase-free water and stored at -20 °C. Randomly selected samples were checked for inhibition of the RT reaction using an RNA internal amplification control. No inhibition was detected (results not shown).

The qPCR was done on an Agilent Mx3005P qPCR System (Agilent Technologies). A qPCR master mix was made for each reaction with 12.5 μL 2× GoTaq Master Mix (Promega), 0.5 μL of 10 μM forward and reverse primer (P009/P010) (Table 1), 0.125 μL of 5 μM *Taqman* probe (Table 1), 1.25 μL 50 mM MgCl_2_ and made up to 20 μL with RNase-free water. The primers and probe were produced by Metabion (Metabion International) and were described previously by Dye et al. [19].

**Table 1:** Primer and probe sequences used in this study

**Name**	**Use**	**Sequence (5’- 3’)**	**Position in FCoV (strain 79-1146**^**1**^**) genome**	**Position in FCoV (strain C1Je**^**2**^**) genome**
P009	qPCR forward primer	AGCAACTACTGCCACRGGAT	26655..26674	
P010	qPCR reverse primer	GGAAGGTTCATCTCCCCAGT	26826..26807	
*Taqman-P1*	FCoV qPCR fluorescent probe	FAM-AATGGCCACACAGGGA	26781..26802	
		CAACGC-BHQ-1		
F614	Forward pyrosequencing primer	GCHCARTATTAYAATGGCAT		23436..23460
		AATGG		
R766	Biotinylated reverse pyrosequencing primer	BIO-AAGYCTRGCYTGYACT		23588..23568
		TGCAT		
S680	Pyrosequencing primer	ACAGCCTCDTTAATAGGVGG		23502..23524
		ATG		
C4	Positive control oligonucleotide	GTAAAGCCRTAGGAGATCGA	Primer does not target FCoV sequence	Primer does not target FCoV sequence
		CATGTAGTTACACTGATGAG		
		TCGATCTCC		

^1^Accession number DQ010921.

^2^Accession number DQ848678.

One-tenth of the randomly primed cDNA (5 μL) was added to the PCR master mix. The reaction plate was heat sealed and the following thermal profile was used: 95 °C for 2 min, 40 cycles of 95 °C for 15 s, 55 °C for 15 s and 72 °C for 15 s. Fluorescence was detected at 520 nm during the extension phase. Feline CoV cDNA was used as a positive control and RNase-free water as a negative control.

Reactions that failed to reach the threshold cycle (Ct) value by cycle 40 were deemed to be negative. A Ct value of 40 was assigned a relative copy number of 1 [21,22], and the following equation, which takes into account the 96% efficiency of the qRT-PCR assay [19], was used to calculate the relative copy number of each qRT-PCR positive sample: 1.96 ^(40-Ct value)^.

### Pyrosequencing

All samples that were positive by FCoV qRT-PCR underwent conventional PCR to amplify a 153 base-pair DNA fragment encompassing position 1058 in the S protein gene. PCR was done using a MJ Mini Gradient Thermal Cycler. Briefly, for each reaction, a PCR mix was made that included 12.5 μL 2× GoTaq Master Mix, 0.5 μL of 10 μM forward and reverse primer (F614/R766) (Table 1), 2 μL of randomly primed cDNA reaction products and water to a volume of 25 μL. The following thermal profile was used; 95 °C for 2 min, 40 cycles of 95 °C for 15 s, 52 °C for 20 s and 72 °C for 20 s, before being held at 4 °C. The PCR products were used for the pyrosequencing reaction or stored at -20 °C until required. Samples that failed to produce definitive sequence data were pyrosequenced, following repeat amplification using the same PCR protocol with 50 cycles of amplification.

Single strand sequencing templates were produced by binding the biotinylated PCR product to streptavidin-coated Sepharose beads (Fisher), followed by chemical denaturation and neutralisation. For each sample, the following mix was prepared; 2 μL streptavidin beads, 40 μL PyroMark binding buffer (Qiagen) (pH 7.6 containing 10 mM Tris-HCl, 2 M NaCl, 1 mM EDTA, 0.1% Tween 20) made up to 55 μL with water. The bead mixture was added to the PCR product and shaken at 1400 rpm for 10 min. The sequencing primer mix contained 0.75 μL 10 μM sequencing primer (Table 1) and 24.25 μL PyroMark annealing buffer (Qiagen) (20 mM Tris-OAc, 5 mM Mg-OAc pH 7.6) for each sample. The control oligonucleotide (a self-sequencing oligonucleotide) mix contained 1 μL 10 μM oligonucleotide C4 and 24 μL annealing buffer. Twenty five microlitres of the sequencing primer mix was added to the appropriate wells of a pyrosequencing plate, 25 μL of control oligonucleotide was added to one well, and the plate was placed on the pyrosequencing workstation.

The pyrosequencing workstation was prepared with trays containing wash buffer (10 mM Tris-OAc pH 7.6), denaturing buffer (0.2 M NaOH), 70% ethanol and distilled water. The streptavidin bead bound PCR product was taken up using a vacuum pump and the pyrosequencing bead collector. The bead collector was then placed in the 70% ethanol for 5 s, in the denaturing buffer for 5 s and in the wash buffer for 10 s. After turning off the vacuum, the bead collector was placed in the pyrosequencing plate and agitated for 30 s to dislodge the beads. The pyrosequencing plate was heated on a plate holder at 80 °C for 2 min, before being placed into the PyroMark Q24 (Qiagen) and left to cool for 5 min.

While the plate was cooling, the pyrosequencing cartridge was prepared. PyroMark Gold Q24 enzyme, substrate and dNTPs (Qiagen) were added into the appropriate wells of the cartridge. Volumes were as outlined by the PyroMark Q24 software. During the experimental set up, the dispensation order of the nucleotides was defined as; CGCTCATG. The cartridge was placed into the PyroMark Q24 instrument and the protocol run.

All primers used in the pyrosequencing assay were designed using a combination of PyroMark assay design software (Qiagen), Primer 3’ software [23] and MFold [24], and were made by Eurofins (MWG Operon) (Table 1). The primer positions were based on those used by Chang et al. [13], and numbered according to the FCoV C1Je genome [GenBank:DQ848678]. Degeneracies were added to the primers, and the location of the primers optimised, based upon a sequence alignment comprised of all available type I FCoV genomes (data not shown).

### Sanger sequencing

Conventional PCR to amplify a 153 base-pair DNA fragment encompassing position 1060 in the S protein gene was carried out as described above, (see pyrosequencing methods) on samples that did not show a M1058L substitution in the pyrosequencing assay. The PCR primers (F614/R766) were then used as sequencing primers in a standard Sanger sequencing protocol (Eurofins, MWG Operon).

### Statistical analysis

The FCoV relative copy numbers were entered into a database (Excel 2010, Microsoft) and exported into IBM SPSS Statistics software (version 19.0). The data sets were evaluated for normal distribution using the Kolmogorov*–*Smirnov (K-S) test. Non-normally distributed data were described as median and range (minimum and maximum values). Data evaluating FCoV relative copy numbers in tissue and faecal samples from cats with and without FIP were analysed using a multilevel modelling approach (MLwiN v2.27) [25], to account for the repeated measures within cats, and a non-parametric Mann-Whitney U test. The conclusions drawn from both analyses were in full agreement, so the simpler Mann-Whitney U test analysis is presented here. Relative copy numbers were compared between the FIP and non-FIP samples for tissue and faecal samples combined, for faecal samples only, and for tissue samples only. Significance was assigned at a level of *P* < 0.05.

### Ethic statement

Historical samples were collected with full informed consent from owners that samples could be used for research purposes. The project has been approved under ethical review by the University of Bristol Animal Welfare and Ethical Review Board (VIN/14/013).

## Results

A total of 112 samples were analysed and full details of the samples and results are shown in Table 2. In cats with FIP, the diagnosis was confirmed by histopathology and the demonstration of FCoV antigen within macrophages in FIP lesions by IHC. In cats without FIP, the diagnosis was made by histology; neoplasia (e.g. lymphoma, astrocytoma, chemodectoma, or biliary cystadenoma), and inflammatory processes (e.g. chronic lymphoplasmacytic infiltrates of unknown aetiology in liver and kidney, and bronchopneumonia) predominated.

**Table 2:** Ct values of FCoV RNA extracted from clinical samples and the amino acids coded at position 1058 and position 1060 in the FCoV S protein

**Group**	**Cat number**	**Type of sample**	**Ct value**	**Codon at position 1058**
Non FIP	33	Tissue	35.8^a^	UUG (Leu)
			None^b^	Not applicable
			None^c^	Not applicable
Non FIP	38	Faeces	34.1	AUG (Met)
		Tissue	None^d^	Not applicable
			None^e^	Not applicable
Non FIP	41	Faeces	26.8	AUG (Met)
		Tissue	None^b^	Not applicable
			None^f^	Not applicable
Non FIP	48	Faeces	34.2	AUG (Met)
		Tissue	35.7^b^	UUG (Leu)
			35.8^a^	UUG (Leu)
Non FIP	51	Tissue	37.3^f^	UUG (Leu)
Non FIP	52	Faeces	None	Not applicable
		Tissue	None^g^	Not applicable
Non FIP	54	Faeces	None	Not applicable
		Tissue	37.3^h^	UUG (Leu)
			None^i^	Not applicable
			None^b^	Not applicable
			None^j^	Not applicable
			None^c^	Not applicable
			None^d^	Not applicable
Non FIP	56	Tissue	35.9^b^	UUG (Leu)
			None^d^	Not applicable
			None^c^	Not applicable
			28.6^k^	AUG (Met)^1^
Non FIP	57	Faeces	None	Not applicable
		Tissue	31.6^l^	UUG (Leu)
			33.4^b^	UUG (Leu)
			None^c^	Not applicable
Non FIP	59	Faeces	33.6	AUG (Met)
		Tissue	None^b^	Not applicable
Non FIP	60	Faeces	35.6	AUG (Met)/CUG (Leu)
		Tissue	None^b^	Not applicable
Non FIP	63	Tissue	None^m^	Not applicable
Non FIP	65	Faeces	None	Not applicable
		Tissue	None^l^	Not applicable
			None^b^	Not applicable
Non FIP	69	Faeces	26.3	AUG (Met)
		Tissue	None^c^	Not applicable
			None^b^	Not applicable
			None^l^	Not applicable
			None^d^	Not applicable
Non FIP	71	Tissue	None^b^	Not applicable
			None^a^	Not applicable
			None^j^	Not applicable
Non FIP	72	Tissue	None^b^	Not applicable
			None^j^	Not applicable
			None^l^	Not applicable
			None^d^	Not applicable
			None^c^	Not applicable
FIP	26	Tissue	17.1^n^	UUG (Leu)
FIP	27	Tissue	26.2^b^	UUG (Leu)
FIP	28	Faeces	19.3	AUG (Met)
		Tissue	25.5^b^	UUG (Leu)
			26.9^j^	UUG (Leu)
FIP	30	Tissue	29.3^b^	UUG (Leu)
FIP	31	Faeces	22.7	AUG (Met)
		Tissue	22.2^f^	UUG (Leu)
FIP	32	Faeces	21.0	AUG (Met)
		Tissue	17.6^a^	UUG (Leu)
			19.2^l^	UUG (Leu)
			26.2^b^	UUG (Leu)
FIP	34	Tissue	20.4^l^	UUG (Leu)
FIP	35	Tissue	20.5^b^	UUG (Leu)
FIP	36	Tissue	31.1^l^	UUG (Leu)
FIP	37	Faeces	15.8	AUG (Met)
		Tissue	16.2^o^	AUG (Met)^2^
FIP	42	Faeces	19.9	AUG (Met)
		Tissue	20.4^l^	UUG (Leu)
			21.6^b^	UUG (Leu)
FIP	43	Tissue	24.1^a^	CUG (Leu)
FIP	44	Faeces	30.0	AUG (Met)
		Tissue	19.0^p^	UUG (Leu)
FIP	45	Tissue	25.0^q^	UUG (Leu)
			27.9^r^	UUG (Leu)
FIP	46	Faeces	34.2	UUG (Leu)
		Tissue	31.7^l^	UUG (Leu)
FIP	47	Faeces	33.3	UUG (Leu)
		Tissue	19.4^s^	UUG (Leu)
FIP	49	Faeces	30.9	AUG (Met)
		Tissue	21.8^c^	UUG (Leu)
FIP	50	Faeces	None	Not applicable
		Tissue	16.1^c^	UUG (Leu)
FIP	53	Faeces	21.9	AUG (Met)
		Tissue	15.5^o^	UUG (Leu)
FIP	55	Tissue	17.7^c^	CUG (Leu)
			20.2^b^	CUG (Leu)
FIP	58	Faeces	None	Not applicable
		Tissue	None^b^	Not applicable
FIP	61	Faeces	23.1	AUG (Met)
		Tissue	None^b^	Not applicable
FIP	62	Tissue	15.3^b^	UUG (Leu)
			17.8^j^	UUG (Leu)
			18.0^a^	UUG (Leu)
			18.9^d^	UUG (Leu)
			20.7^l^	UUG (Leu)
			30.9^c^	UUG (Leu)
FIP	66	Faeces	25.5	UUG (Leu)
		Tissue	16.7^j^	UUG (Leu)
			24.7^b^	UUG (Leu)
FIP	67	Faeces	31.6	AUG (Met)
FIP	68	Tissue	31.1^t^	UUG (Leu)
			31.3^l^	UUG (Leu)
			33.1^b^	UUG (Leu)
			34.0^j^	UUG (Leu)
			36.9^d^	UUG (Leu)
FIP	70	Faeces	None	Not applicable
		Tissue	23.3^b^	AUG (Met)^1^
			29.3^d^	AUG (Met)^1^
			30.3^l^	AUG (Met)^1^
			31.0^j^	UUG (Leu)

Tissue types are defined by superscripts: ^a^omentum, ^b^liver, ^c^kidney, ^d^lung, ^e^stomach, ^f^lymph node, ^g^cerebrum, ^h^abdominal lymph node, ^i^heart, ^j^spleen, ^k^colon, ^l^mesenteric lymph node, ^m^brain, ^n^mesentery, ^o^pleura, ^p^pericardium, ^q^medulla, ^r^pons, ^s^small intestine, ^t^intestine. The amino acid coded at position 1060 in the S protein of FCoV RNA from tissue samples without the M1058L substitution is defined by the superscripts: ^1^UCU (Ser) and ^2^GCU (Ala).

### Quantitative RT-PCR

A total of 26 faecal samples were analysed by FCoV qRT-PCR, and 19 (73%) were positive. These comprised 13 of 16 (81%) faecal samples from cats with FIP, and 6 of 10 (60%) faecal samples from cats without FIP (Table 2).

A total of 86 tissue samples were analysed by FCoV qRT-PCR, and 52 (60%) were positive. These comprised 43 of 45 (95%) tissue samples from cats with FIP, and 9 of 41 (22%) tissue samples from cats without FIP (Table 2).

Relative FCoV RNA copy numbers were not normally distributed (*P* < 0.001). The relative copy numbers in pooled faecal and tissue samples in the FIP group (median; range: 44 347; 0-16 547 217) were significantly higher (U = 241.0, *P* < 0.001) than in the non-FIP group (0, 0-10 090). When only tissue samples were considered, the relative copy numbers in the FIP group (75 976; 0-16 574 217) were also significantly higher than those in the non-FIP group (0; 0-2 146) (U = 72, *P* < 0.001). Finally, analysis of faecal samples alone showed that the relative copy numbers in the FIP group (9 062; 0-11 819 441) were significantly higher than those in the non-FIP group (34; 0-10 090) (U = 36.5, *P* = 0.02).

### Pyrosequencing

The 19 faecal and 52 tissue samples with positive qRT-PCR results were subjected to the pyrosequencing assay. Of the 19 faecal samples successfully sequenced, 10 were obtained using the 40 cycle pyrosequencing assay, whereas 9 required the 50 cycle assay. Of the 52 tissue samples successfully sequenced, 34 were obtained using the 40 cycle pyrosequencing assay, whereas 18 required the 50 cycle PCR.

#### Faecal samples positive by qRT-PCR

**Figure 1:**
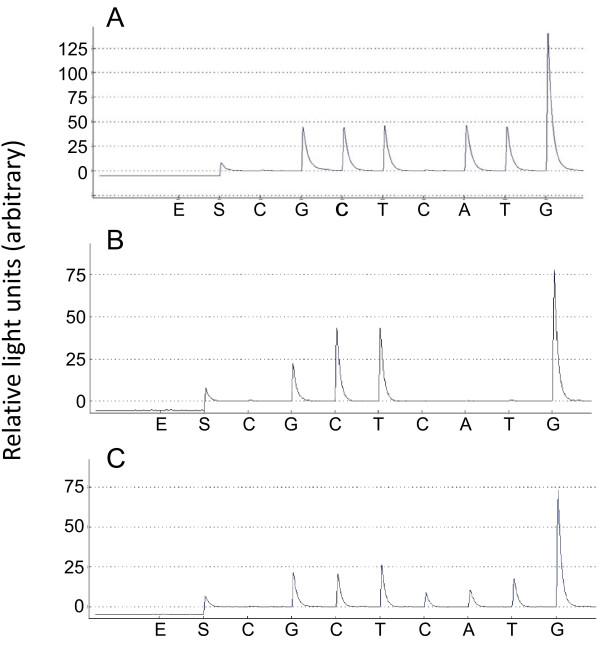
**Representative sequencing pyrograms of codon 1058 in faecal RNA samples. A**: Pyrogram with an ATG (codon = methionine) sequence at position 1058 from cat 32, faeces. The peaks represent a sequence of GCT*ATG*GG. **B**: Pyrogram with a TTG (codon = leucine) sequence at position 1058 from cat 46, faeces. The peaks represent a sequence of GCC*TTG*GG. **C**: Pyrogram with a mixture of ATG (codon = methionine) and CTG (codon = leucine) sequences at position 1058 from cat 60, faeces. The peaks represent a sequence of GCT(*C/A)TG*GG; the relative peak heights suggest that the methionine and leucine encoding RNAs were present in approximately equal amounts. The horizontal axis shows the nucleotide dispensation order. E, enzyme-only control; S, substrate-only control. The initial nucleotide injected **(C)** is irrelevant to the sequence and provides a baseline.

In 15 of the 19 (79%) faecal samples positive by qRT-PCR, a methionine (AUG) codon alone was found at position 1058 (Table 2 and Figure 1A). In 3 of the 19 (16%) samples, a leucine codon (UUG) alone was found at position 1058 (Table 2 and Figure 1B). Additionally, 1 (5%) sample (cat 60, faeces) showed a mixed population of RNA coding for either methionine (AUG) or leucine (CUG) at this position (Table 2 and Figure 1C).

Importantly, methionine and leucine codons were identified in faecal RNA samples from both cats with and without FIP (Table 2). Specifically, a methionine codon was identified in 10 samples from 10 FIP cats, and a leucine codon was identified in 3 samples from 3 FIP cats. Overall, a methionine codon was found in the majority (10/13; 77%) of faecal samples from cats with FIP, but a significant number had a leucine codon (3/13; 23%).

Similarly, a methionine codon was identified in 6 samples from 6 cats without FIP (including one mixed infection), and a leucine codon was identified in 1 sample from 1 cat without FIP (which had the mixed infection). Overall, a methionine codon was found in all 6 faecal samples from cats without FIP, and 1 sample in 1 cat had a leucine codon (1/6; 17%, which also had a methionine as a mixed infection).

#### Tissue samples positive by qRT-PCR

**Figure 2:**
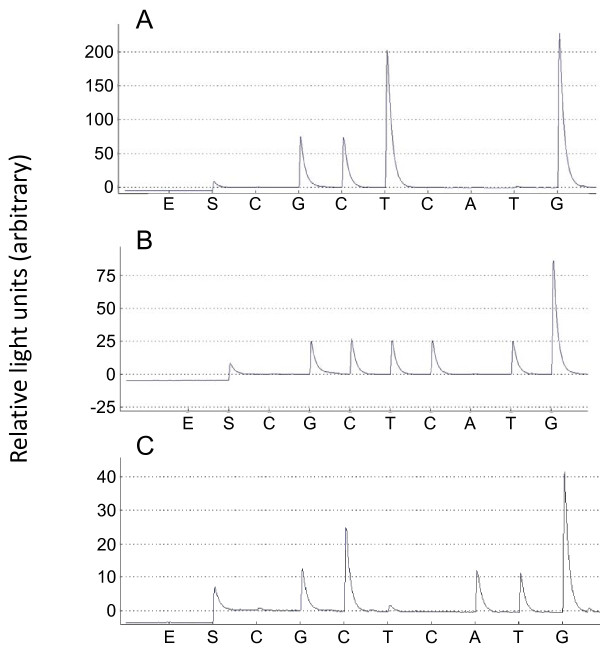
**Representative sequencing pyrograms of codon 1058 in tissue RNA samples. A**: Pyrogram with a TTG (codon = leucine) sequence at position 1058 from cat 47, small intestine. The peaks represent a sequence of GCT*TTG*GG. **B**: Pyrogram with a CTG (codon = leucine) sequence at position 1058 from cat 55, liver. The peaks represent a sequence of GCT*CTG*GG. **C**: Pyrogram with an ATG (codon = methionine) sequence at position 1058 from cat 70, liver. The peaks represent a sequence of GCC*ATG*GG. The horizontal axis shows the nucleotide dispensation order. E, enzyme-only control; S, substrate-only control. The initial nucleotide injected **(C)** is irrelevant to the sequence and provides a baseline.

In 47 of the 52 (90%) tissue samples positive by qRT-PCR, a leucine codon (44 UUG, 3 CUG) alone was found at position 1058 (Table 2 and Figures 2A and 2B). In the remaining 5 of the 52 (10%) tissue samples, a methionine codon (AUG) was found at this position (Table 2 and Figure 2C). No mixed infections were found in tissue samples.

Importantly, leucine and methionine codons were identified in RNA from tissue samples from both cats with and without FIP (Table 2). Specifically, a leucine codon was identified in 39 samples from 23 FIP cats, and a methionine codon was identified in 4 samples from 2 FIP cats. Overall, a leucine codon was found in the majority (39/43; 91%) of FIP tissue samples, but a significant number had a methionine codon (4/43; 9%). Similarly, a leucine codon was identified in 8 samples from 6 cats without FIP, and a methionine codon was identified in 1 sample from 1 cat without FIP. Overall, a leucine codon was found in the majority (8/9; 89%) of tissue samples from cats without FIP, with a minority (1/9; 11%) having a methionine codon. In one specific case (cat 70), we noted that the sample from one tissue (spleen) contained a leucine codon, whereas samples from three other tissues (liver, lung and mesenteric lymph node) all contained a methionine codon.

### Sanger sequencing of substitution S1060A

Conventional RT-PCR amplification products of RNA from 5 tissue samples that did not contain the M1058L substitution (from cats 37, 56 and 70) were analysed by Sanger sequencing for the S1060A substitution. One of the 5 samples (cat 37, pleura) showed an alanine codon at position 1060. The remaining four samples (1 from cat 56 and three from cat 70) all showed a serine codon at this position (Table 2).

## Discussion

The most important finding in this study is that the M1058L substitution in the FCoV S protein does not correlate with FIP disease phenotype, as was proposed by Chang et al. [13]. We reach this conclusion because of two observations. First, although a leucine codon was found in the majority (91%) of tissue samples with FIP lesions, a leucine codon was also found in the majority (89%) of non-FIP tissue samples. Second, a significant number (9%) of FIP tissue samples had a methionine codon at this position. Tissue samples from naturally FCoV infected cats without FIP have not been previously evaluated and provide an important insight into FCoV infection in the absence of FIP. We believe that the M1058L substitution is more likely to be a marker of systemic FCoV infection, as opposed to a marker of FIP or the development of disease. As our tissue samples were collected post-mortem, we cannot exclude the possibility that the 6 cats without FIP (8 samples, from cats 33, 49, 51, 54, 56 and 57) and the leucine codon at position 1058, would have gone on to develop FIP if they had not been euthanized due to other reasons. However, histopathological changes consistent with FIP were absent in all 6 cats, and IHC did not identify FCoV antigen.

The finding that the majority of tissue samples from both cats with and without FIP have a leucine codon at position 1058 does not challenge the idea that systemic spread of FCoV is an important step in the development of FIP. Indeed, the latter is supported by the findings of our study, since FCoV RNA was found in a far greater proportion of tissue samples with FIP lesions (95%) than tissue samples from cats without FIP (22%), and, in those samples that were qRT-PCR positive, significantly higher FCoV relative copy numbers were found in the FIP samples, as has been found in a previous study in naturally infected cats [26]. However, as sampling in our study took place post-mortem, it could also be argued that the elevated FCoV levels in FIP tissues were a consequence of the massive immunological dysregulation that results from the disease, rather than a contributing factor towards the development of disease.

Another viewpoint is that the low levels of FCoV RNA from the tissues of cats without FIP are a result of FCoV infecting only fully differentiated macrophages and monocytes, among which are tissue-specific macrophages. This view is supported by our results as IHC, a detection method of low sensitivity, did not detect FCoV antigen anywhere in the PCR-positive tissues from cats without FIP, providing further evidence of low level viral infection either of tissue macrophages (in persistently infected animals) or in monocytes that were in vessels in the respective organ at the time of sampling [27]. These findings are also in accordance with recent in vivo and in vitro studies that showed only the virulent form of FCoV can effectively and sustainably replicate in monocytes [9,28,29].

The M1058L substitution was not the only S protein substitution that was proposed by Chang et al. [13] to correlate with the FIP disease phenotype. They also showed that a second substitution, S1060A, distinguished a further 4% of FIP from non-FIP associated FCoVs. We confirmed this result in so far as RNA obtained from one of five tissue samples that did not show the M1058L substitution showed the S1060A substitution.

We believe that, as was proposed by Chang et al. [13], changes such as the M1058L and S1060A substitutions, and potentially others, could be representative of a class of mutations that influence the fusogenic activity of the FCoV S protein and, as such, deserve particular attention with regard to the pathogenesis of FIP [13]. It is noteworthy that the substitutions identified by Licitra et al. [15] that are proposed to distinguish between FCoVs from animals with and without FIP are also suggested to have an effect upon the fusogenic activity of the S protein.

With regard to faecal samples, our study found that an unexpectedly high percentage (81%) of faecal samples from cats with FIP were FCoV qRT-PCR positive and their relative copy numbers were significantly higher than those of faecal samples from cats without FIP. Moreover, the majority (77%) of FCoV RNA sequences in faecal samples from cats with FIP had a methionine codon at position 1058 in the FCoV S protein gene, suggesting that these animals were shedding an enteric form of the virus. It seems reasonable to suggest that these cats were infected with an enteric, and a systemic, virulent form of FCoV. Whether one form was derived from the other following a single infection or whether these cats were infected twice with different FCoVs cannot be determined. It has been proposed that the severe immune dysregulation in cats with end-stage FIP might create an opportunity for super-infection by enteric FCoV circulating in surrounding carriers [17]. More interestingly, a smaller but significant proportion of faecal samples from cats with FIP (23%) provided FCoV RNA samples that encoded leucine at position 1058. The current model of FIP pathogenesis proposes that once the enteric form of the virus has mutated to a virulent form, it is generally no longer horizontally transmitted via the faeces [12,16]. This view has been challenged [30], and the current results also suggest that a systemic form of the virus can be found in the faeces of FIP cats. However, we accept that this does not mean that excreted virus is necessarily able to infect further cats by the enteric route, as has recently been shown in some experimental studies [16]. Further research is necessary to resolve these issues.

In contrast to the pattern shown by the analysis of faecal samples from cats with FIP, the analysis of the faecal samples from cats without FIP seemed more straight-forward. All “non-FIP” faecal samples that were FCoV qRT-PCR positive encoded methionine at position 1058, indicative of infection with the enteric form of the virus. Also, as found in our study, a shedding proportion of 60% by cats without FIP is not unexpected [12,31]. An interesting faecal sample from a cat without FIP (cat 60) showed a mixed population of RNAs encoding for either methionine or leucine at position 1058. There was no evidence of FCoV RNA in the single tissue sample taken from cat 60, and, therefore, one interpretation could be that the M1058L substitution in the faecal sample was a relatively recent event and the virus had not yet spread systemically. However, this interpretation has to be considered as tentative because we have observed examples of negative qRT-PCR results in tissue samples from cats that were clearly FCoV infected due to their FIP grouping; cat 58, liver; cat 61, liver. As histopathology results and IHC for these two liver samples showed changes consistent with FIP, these negative qRT-PCR results are likely to have arisen due to an absence of FCoV in the particular samples taken for molecular analysis, as a variable distribution of FCoV in affected tissues has been reported [18].

Our study importantly also demonstrated that a PCR-based pyrosequencing [32] approach is a rapid and accurate method to identify single nucleotide differences at a specific position within a DNA fragment, or in our case a viral genome. However, it has some limitations. Some samples required 50, rather than 40, cycles of PCR amplification to generate adequate amounts of DNA for sequencing. This was especially true for PCR products generated from samples that contained low amounts of viral RNA, e.g. tissue samples from cats without FIP. There were also several samples which, despite containing quantities of viral RNA measurable by qRT-PCR, did not produce sufficient PCR products for pyrosequencing, even after 50 amplification cycles. These samples were excluded from the results as they did not contribute any additional sequence data to the study. However, one explanation may be that, despite the degeneracy of the primers used (F614/R766), differences in the viral primer binding sites may have precluded efficient amplification in these samples.

In summary, we have used a pyrosequencing assay to determine the distribution of a specific M1058L substitution in the S protein of FCoV RNA obtained from a large number of post-mortem tissue and faecal samples from cats with and without FIP. Additionally, Sanger sequencing was used to determine whether the S1060A substitution was present in tissue samples that did not contain the M1058L substitution. This represents the first study that compares similar samples from cats with and without FIP with regard to the viral phenotype. Our results contribute to a better understanding of FCoV genomic mutations and how they may, or may not, be used as markers of the virus phenotype. The results also make clear that the relationship between the viral genotype and the development of FIP is complex. We are currently using an approach that involves the deep sequencing of complete FCoV genomes in clinical samples, in order to throw further light on this relationship.

## Competing interests

The authors declare that they have no competing interests.

## Authors’ contributions

EP carried out the in vitro experimental work. EP, ST, MJD and RH obtained samples. MJD carried out the histopathological analysis. AK undertook the immunohistochemical examinations. CRH supervised the design of the qRT-PCR and pyrosequencing assays. EP and ST carried out the statistical analysis. EP, ST, CRH and SGS participated in the design of the study, analysed the results and drafted the manuscript. All authors contributed to the writing of the manuscript and approved the final manuscript.

## References

[B1] Sparkes AH Gruffydd-Jones TJ Harbour DA Feline coronavirus antibodies in UK cats Vet Rec 1992 131 223 224 1332241 10.1136/vr.131.10.223-a

[B2] Pedersen NC Serologic studies of naturally occurring feline infectious peritonitis Am J Vet Res 1976 37 1449 1453 793459

[B3] Guan Y Zheng BJ He YQ Liu XL Zhuang ZX Cheung CL Luo SW Li PH Zhang LJ Guan YJ Butt KM Wong KL Chan KW Lim W Shortridge KF Yuen KY Peiris JS Poon LL Isolation and characterization of viruses related to the SARS coronavirus from animals in southern China Science 2003 302 276 278 10.1126/science.1087139 12958366

[B4] Addie D Belak S Boucraut-Baralon C Egberink H Frymus T Gruffydd-Jones T Hartmann K Hosie MJ Lloret A Lutz H Marsilio F Pennisi MG Radford AD Thiry E Truyen U Horzinek MC Feline infectious peritonitis. ABCD guidelines on prevention and management J Feline Med Surg 2009 11 594 604 10.1016/j.jfms.2009.05.008 19481039 PMC7129471

[B5] Addie DD Clustering of feline coronaviruses in multicat households Vet J 2000 159 8 9 10.1053/tvjl.1999.0429 10640407 PMC7172047

[B6] Pedersen NC Sato R Foley JE Poland AM Common virus infections in cats, before and after being placed in shelters, with emphasis on feline enteric coronavirus J Feline Med Surg 2004 6 83 88 10.1016/j.jfms.2003.08.008 15123152 PMC7128562

[B7] Addie DD Jarrett O A study of naturally occurring feline coronavirus infections in kittens Vet Rec 1992 130 133 137 10.1136/vr.130.7.133 1313617

[B8] Regan AD Cohen RD Whittaker GR Activation of p38 MAPK by feline infectious peritonitis virus regulates pro-inflammatory cytokine production in primary blood-derived feline mononuclear cells Virology 2009 384 135 143 10.1016/j.virol.2008.11.006 19058829 PMC7103373

[B9] Dewerchin HL Cornelissen E Nauwynck HJ Replication of feline coronaviruses in peripheral blood monocytes Arch Virol 2005 150 2483 2500 10.1007/s00705-005-0598-6 16052283 PMC7086860

[B10] Kipar A May H Menger S Weber M Leukert W Reinacher M Morphologic features and development of granulomatous vasculitis in feline infectious peritonitis Vet Pathol 2005 42 321 330 10.1354/vp.42-3-321 15872378

[B11] Olyslaegers DA Dedeurwaerder A Desmarets LM Vermeulen BL Dewerchin HL Nauwynck HJ Altered expression of adhesion molecules on peripheral blood leukocytes in feline infectious peritonitis Vet Microbiol 2013 166 438 449 10.1016/j.vetmic.2013.06.027 23910523 PMC7117481

[B12] Pedersen NC A review of feline infectious peritonitis virus infection: 1963-2008 J Feline Med Surg 2009 11 225 258 10.1016/j.jfms.2008.09.008 19254859 PMC7129802

[B13] Chang HW Egberink HF Halpin R Spiro DJ Rottier PJ Spike protein fusion peptide and feline coronavirus virulence Emerg Infect Dis 2012 18 1089 1095 10.3201/eid1807.120143 22709821 PMC3376813

[B14] Bosch BJ van der Zee R de Haan CA Rottier PJ The coronavirus spike protein is a class I virus fusion protein: structural and functional characterization of the fusion core complex J Virol 2003 77 8801 8811 10.1128/JVI.77.16.8801-8811.2003 12885899 PMC167208

[B15] Licitra BN Millet JK Regan AD Hamilton BS Rinaldi VD Duhamel GE Whittaker GR Mutation in spike protein cleavage site and pathogenesis of feline coronavirus Emerg Infect Dis 2013 19 1066 1073 10.3201/eid1907.121094 23763835 PMC3713968

[B16] Pedersen NC Liu H Scarlett J Leutenegger CM Golovko L Kennedy H Kamal FM Feline infectious peritonitis: role of the feline coronavirus 3c gene in intestinal tropism and pathogenicity based upon isolates from resident and adopted shelter cats Virus Res 2012 165 17 28 10.1016/j.virusres.2011.12.020 22280883 PMC7114484

[B17] Chang HW de Groot RJ Egberink HF Rottier PJ Feline infectious peritonitis: insights into feline coronavirus pathobiogenesis and epidemiology based on genetic analysis of the viral 3c gene J Gen Virol 2010 91 415 420 10.1099/vir.0.016485-0 19889934

[B18] Kipar A Bellmann S Kremendahl J Kohler K Reinacher M Cellular composition, coronavirus antigen expression and production of specific antibodies in lesions in feline infectious peritonitis Vet Immunol Immunopathol 1998 65 243 257 10.1016/S0165-2427(98)00158-5 9839877 PMC7119884

[B19] Dye C Helps CR Siddell SG Evaluation of real-time RT-PCR for the quantification of FCoV shedding in the faeces of domestic cats J Feline Med Surg 2008 10 167 174 10.1016/j.jfms.2007.10.010 18243744 PMC2582154

[B20] Dye C Siddell SG Genomic RNA sequence of feline coronavirus strain FCoV C1Je J Feline Med Surg 2007 9 202 213 10.1016/j.jfms.2006.12.002 17363313 PMC2582377

[B21] Barker EN Tasker S Day MJ Warman SM Woolley K Birtles R Georges KC Ezeokoli CD Newaj-Fyzul A Campbell MD Sparagano OA Cleaveland S Helps CR Development and use of real-time PCR to detect and quantify Mycoplasma haemocanis and “Candidatus Mycoplasma haematoparvum” in dogs Vet Microbiol 2010 140 167 170 10.1016/j.vetmic.2009.07.006 19646827 PMC2805721

[B22] Helps C Reeves N Tasker S Harbour D Use of real-time quantitative PCR to detect Chlamydophila felis infection J Clin Microbiol 2001 39 2675 2676 10.1128/JCM.39.7.2675-2676.2001 11427593 PMC88209

[B23] Rozen S Skaletsky H Primer3 on the WWW for general users and for biologist programmers Methods Mol Biol 2000 132 365 386 10547847 10.1385/1-59259-192-2:365

[B24] Zuker M Mfold web server for nucleic acid folding and hybridization prediction Nucleic Acids Res 2003 31 3406 3415 10.1093/nar/gkg595 12824337 PMC169194

[B25] Rasbash J Charlton C Browne WJ Healy M Cameron B MLwiN Version 2.1 Centre for Multilevel Modelling, University of Bristol 2009

[B26] Kipar A Baptiste K Barth A Reinacher M Natural FCoV infection: cats with FIP exhibit significantly higher viral loads than healthy infected cats J Feline Med Surg 2006 8 69 72 10.1016/j.jfms.2005.07.002 16213766 PMC7129897

[B27] Kipar A Meli ML Baptiste KE Bowker LJ Lutz H Sites of feline coronavirus persistence in healthy cats J Gen Virol 2010 91 1698 1707 10.1099/vir.0.020214-0 20237226

[B28] Rottier PJ Nakamura K Schellen P Volders H Haijema BJ Acquisition of macrophage tropism during the pathogenesis of feline infectious peritonitis is determined by mutations in the feline coronavirus spike protein J Virol 2005 79 14122 14130 10.1128/JVI.79.22.14122-14130.2005 16254347 PMC1280227

[B29] Simons FA Vennema H Rofina JE Pol JM Horzinek MC Rottier PJ Egberink HF A mRNA PCR for the diagnosis of feline infectious peritonitis J Virol Methods 2005 124 111 116 10.1016/j.jviromet.2004.11.012 15664058 PMC7112896

[B30] Wang YT Su BL Hsieh LE Chueh LL An outbreak of feline infectious peritonitis in a Taiwanese shelter: epidemiologic and molecular evidence for horizontal transmission of a novel type II feline coronavirus Vet Res 2013 44 57 10.1186/1297-9716-44-57 23865689 PMC3720556

[B31] Addie DD Jarrett O Use of a reverse-transcriptase polymerase chain reaction for monitoring the shedding of feline coronavirus by healthy cats Vet Rec 2001 148 649 653 10.1136/vr.148.21.649 11400984

[B32] Fakhrai-Rad H Pourmand N Ronaghi M Pyrosequencing: an accurate detection platform for single nucleotide polymorphisms Hum Mutat 2002 19 479 485 10.1002/humu.10078 11968080

